# Monkey finger mycology? First case of otomycosis externa caused by *Trichophyton simii* after encounter with a monkey

**DOI:** 10.1016/j.mmcr.2022.06.001

**Published:** 2022-06-09

**Authors:** Sigmund Krajden, Richard C. Summerbell, Aswani Datt, Mike Hawke, James Scott

**Affiliations:** a St. Joseph's Health Centre, 30 The Queensway, Toronto, ON, M6R 1B5, Canada; b Sporometrics, 219 Dufferin St #20c, Toronto, ON, M6K 3J1, Canada; c Dalla Lana School of Public Health, 155 College St Room 500, Toronto, ON, M5T 3M7, Canada; d Faculty of Medicine, University of Toronto, M5S 1A8, Canada

**Keywords:** Otomycosis, Trichophyton simii, Otitis, Simian, Travel history

## Abstract

Unilateral ear pain, ear canal blockage and reduced hearing in an 18-year-old Canadian male who had travelled to India revealed, on examination of a swab, secretions bearing unusual fungal filaments visually suggestive of dermatophyte elements. Culture yielded *Trichophyton simii*, an unusual skin infecting species with a worldwide distribution but most often seen from India. The patient recalled swimming in the Ganges River but also had had his ear manipulated by a street monkey.

## 1 Introduction

Otomycosis is a fungal infection of the external auditory canal and can occur as a primary infection or can develop along with a bacterial otitis externa, usually as a result of antibiotic treatment. In cases not influenced by antibiotic treatment, introduction of a foreign object or soil or debris often precedes infection, especially in young children who are the most commonly affected group. The causative agents are mostly members of the genera *Aspergillus* and *Candida*, especially the *A. niger* complex*, C. albicans*, *A. flavus* and *A. fumigatus*, with *Candida* species predominating in immunocompromised patients and *Aspergillus* in the immunocompetent [1,2].

Members of the dermatophytes, normally seen from infected skin and nails, have seldom been implicated in otomycosis, and when they have, no case details have been given. Reports include three involving morphologically identified *Trichophyton mentagrophytes* complex members [1,3,4], and one involving *T. rubrum* [1]. *Trichophyton simii* is a dermatophyte closely related to the *T. mentagrophytes* complex. It was long thought to be endemic to the Indian subcontinent but is now suspected to have a cosmopolitan distribution, although it is unusual or rare in most localities [5]. Records exist from Belgium, France, Brazil, Iran, Saudi Arabia, Ivory Coast, Argentina and the U.S.A. The species is mainly known from tinea of the nails and glabrous skin. It has not been reported to cause otomycosis.

We herein wish to report a case of otomycosis seen in Canada but featuring an imported *T. simii* isolate from India.

## 2 Case

The patient was an 18-year-old male in excellent health who visited family in the city of Haridwar, Uttar Pradesh State, India, during the summer. He noted that he swam in the Ganges River, possibly exposing his external acoustic meatus to the highly eutrophic waters [6], but recalled as well that during his travels, while he was driving a car and stopped at a crossroads, he had had a monkey enter through the open window and manipulate his right ear while confronting him momentarily before exiting again. He was uncertain about which monkey species was involved.

The patient had had a long-standing practice of assiduously removing the cerumen daily from his ear canals with a cotton swab.

On returning to Canada, he described his right ear as being swollen and painful, and yielding a liquid discharge. He experienced diminished hearing on the right side.

**Fig. 1:**
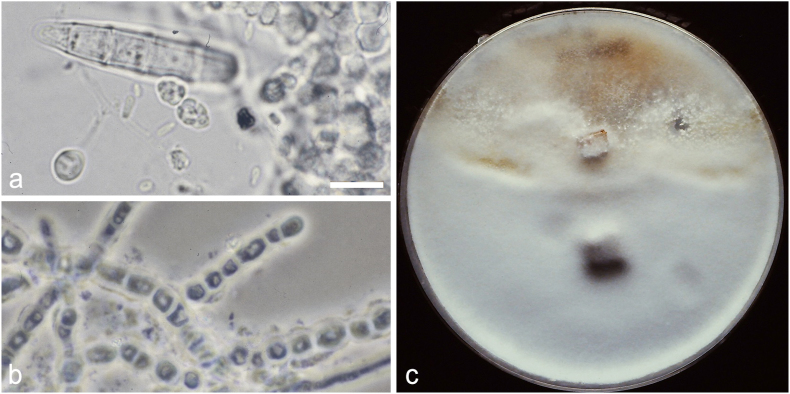
*Trichophyton simii* from ear canal infection. Scale bar 10 μm applies a-b. a. Mount from culture showing macro- and microconidia and 8-spored asci adjacent to ossiform cells composing ascomatal filaments. Structures from 14-day-old colony on Weitzman & Silva-Hutner's medium, 25 C. b. Direct microscopy of ear canal secretions showing hyphae with block-shaped cells suggestive of dermatophyte substrate arthroconidia. c. Culture showing ascomata (small ball-shaped yellowish tufts) arising on confrontation line between case isolate (top) and mating tester stock (bottom).

His family physician prescribed topical antibiotics but these yielded no benefit. An otolaryngologist was consulted who carried out debridement and noted that a white waxy paste-like material was present deep in the bony ear canal. The skin was erythematous and irritated. A sample was sent for microscopy and culture. A pure growth was obtained of numerous colonies morphologically compatible with *Trichophyton simii* (Fig. 1a). The isolate could be unequivocally confirmed as a member of that species by a fully compatible mating reaction with one of two tester isolates (UAMH 2943 and 2944) leading to production of rounded, fuzzy sexual fruiting bodies (ascomata) (Fig. 1c) and fertile sexual ascospores (Fig. 1a). The ear secretions were seen microscopically to be invested with unusual fungal filaments (Fig. 1b) showing a beaded structure consistent with dermatophytic substrate arthroconidia [7]. Pure Canesten ® (clotrimazole) was instilled in the ear canal and a follow-up visit after 14 days revealed a pristine ear canal. The etiologic isolate was not preserved.

## 3 Discussion

*Trichophyton simii* is an infrequently seen fungus that has been reported as an etiologic agent of tinea corporis, tinea cruris, tinea capitis and onychomycosis. This fungus has most frequently been seen in the Indian subcontinent, where it has been isolated from the soil as well as from infected animals and humans. For some years, the preponderance of known isolates was from monkeys, as implied by the epithet *‘simii*,’ but in time, a regular association with poultry and poultry farm work was also noticed [5,8,9]. With isolates from disparate sources like soil in the Ivory Coast [5] and cattle in southern India [10] the species has formed a pattern most consistent with geophilic ecological classification, i.e., breaking down hair, feathers and other keratinous remains in contact with soil, but it cannot be ruled out as a possible zoophile connected with soil-dwelling animals.

Although dermatophytes do not inhabit aqueous habitats, their dry spores and their somewhat water-repellent substrates, like bits of feather, may float on water. Thus, there is no clear resolution as to which event experienced by the patient - swimming in the Ganges or having his ear touched by a wild monkey - was the more likely source of his aural *T. simii* inoculum. It remains possible that his infection was not caused by either event, and was just acquired from normal exposure to background dust, but its novelty suggests an unusual precipitating event. If the monkey invading the car had recently been grooming *T. simii* lesions on itself or others, or had contacted contaminated soil, its fingers may have been replete with inoculum.

*T. simii* predominantly uses integumental proteins as a nutrient source, and some components of otic cerumen would likely be metabolically unavailable to it, but possibly there was sufficient shed and deteriorated skin scale material in the waxy crust seen in direct microscopy to support growth. Impacted ear wax is known to be circa 60% keratin derived from sheets of shed skin cells [11]. As the patient in this case regularly attempted ear wax removal, available substrate might have been scarce or patchy. Moreover, 12–20% of cerumen consists of the sterol precursor squalene, which has been found to inhibit dermatophyte growth [12,13]. Partial removal of cerumen may have facilitated dermatophyte growth on underlying epidermis. It is likely, in any case, that the marginal nature of the habitat for this species contributed to the rapidity of its cure with topical application of clotrimazole. It would be of interest if additional cases of dermatophyte involvement in otomycosis were studied with attention to the unusual aspects of the infections.

## Declaration of competing interest

None.
